# Expertise in clinical pathology: combining the visual and cognitive perspective

**DOI:** 10.1007/s10459-015-9589-x

**Published:** 2015-02-13

**Authors:** Thomas Jaarsma, Halszka Jarodzka, Marius Nap, Jeroen J. G. van Merriënboer, Henny P. A. Boshuizen

**Affiliations:** 1 Welten Institute, Open University of The Netherlands, P.O. Box 2960, 6401 DL Heerlen, The Netherlands; 2 Pathology Department, Atrium Medical Center, Heerlen, The Netherlands; 3 Department of Educational Development and Research, Maastricht University, Maastricht, The Netherlands; 4 University of Turku, Turku, Finland

**Keywords:** Clinical pathology, Cognitive expertise, Visual expertise, Eye tracking, Microscopic images

## Abstract

Expertise studies in the medical domain often focus on either visual or cognitive aspects of expertise. As a result, characteristics of expert behaviour are often described as either cognitive or visual abilities. This study focuses on both aspects of expertise and analyses them along three overarching constructs: (1) encapsulations, (2) efficiency, and (3) hypothesis testing. This study was carried out among clinical pathologists performing an authentic task: diagnosing microscopic slides. Participants were 13 clinical pathologists (experts), 12 residents in pathology (intermediates), and 13 medical students (novices). They all diagnosed seven cases in a virtual microscope and gave post hoc explanations for their diagnoses. The collected data included eye movements, microscope navigation, and verbal protocols. Results showed that experts used lower magnifications and verbalized their findings as diagnoses. Also, their diagnostic paths were more efficient, including fewer microscope movements and shorter reasoning chains. Experts entered relevant areas later in their diagnostic process, and visited fewer of them. Intermediates used relatively high magnifications and based their diagnoses on specific abnormalities. Also, they took longer to reach their diagnosis and checked more relevant areas. Novices searched in detail, described findings by their appearances, and uttered long reasoning chains. These results indicate that overarching constructs can justly be identified: encapsulations and efficiency are apparent in both visual and cognitive aspects of expertise.

## Introduction

Expertise studies in the medical domain often focus on either cognitive or visual aspects of medical expertise. The availability of eye-tracking methodology facilitated the identification of characteristics of expert viewing, such as an increased visual span and the ability to switch between global and focal viewing (Reingold and Sheridan 2011). Running parallel to this visual perspective, medical expertise has been studied from a cognitive viewpoint. Boshuizen and Schmidt (1992, 2008) proposed a three-stage model of medical expertise development, centred around the concepts of knowledge encapsulation and illness scripts. However, for some medical specialties, such as those that rely on images for diagnoses, expertise is expressed in both visual and cognitive aspects. The aim of this study is to identify characteristics of different levels of visual medical expertise, expressed in both visual and cognitive aspects of expertise.

The domain of this study is clinical pathology, the medical specialty that is concerned with the microscopic study of tissue and cells to diagnose the nature of the disease. Some of the previous studies on clinical pathology expertise (Krupinski et al. 2006, 2013; Tiersma et al. 2003) had a relatively low authenticity of the experimental task: participants diagnosed fixed microscopic images instead of zoomable microscopic images. In other studies where zoomable images were used, data were not analysed quantitatively (Mello-Thoms et al. 2012; Treanor et al. 2009). These methodological choices leave a relevant aspect of clinical pathologists’ behaviour out of the equation: zooming and panning within microscopic images. Therefore, this study uses an authentic, unrestricted task. To reach the aim mentioned above, data will be collected on both aspects of expertise and analysed in a combined manner.

In this introduction, we will first discuss the findings on visual and cognitive aspects of expertise separately and consequently identify links between them. We will thereby speak of ‘visual expertise’ and ‘cognitive expertise’ to refer to both aspects of expertise, as these concepts are often used in literature. However, it is important to note that we do not perceive them as separate phenomena, but rather two sides of the same phenomenon. After aligning the results from literature, overarching constructs will be identified and hypotheses will be formed for each construct. These hypotheses will include specific predictions on measures from both visual and cognitive expertise.

In their overview of visual expertise research, Reingold and Sheridan (2011, p. 533) attribute the superior performance of experts to their “superior encoding of domain related configurations”. An important component of this trait is global processing, which means that large parts of an image can be processed simultaneously. This is facilitated by the relatively large visual span of experts, which results in an efficient scan path through an image (ibid.). Such an efficient scan path consists of relatively few and long fixations, with long distances between them (i.e., longer saccades). In addition, as their overview of eye movement studies in the medical domain points out, it is shown in many studies that experts fixate on relevant parts of the image (i.e., abnormalities) faster, and spend more time looking at them than non-experts.

Although Reingold and Sheridan’s overview of expert performance is largely based on studies in radiology, these results correspond with those obtained in studies on the viewing behaviour of clinical pathologists. The enlarged visual span, as well as longer saccades and shorter times on task (i.e., an efficient scan path), were also obtained by Krupinski et al. (2006) in a study using static images and collecting eye movement data only. Krupinski and colleagues also found the experts’ tendency to focus on relevant areas. In two other studies, actual microscopic images were investigated, using microscope navigation data instead of eye movements to study visual processing. Treanor et al. (2009) analysed microscope navigation qualitatively and found that the diagnostic paths of experts were ‘cleaner’ than those of residents. They showed fewer revisits to previously visited areas and were less repetitive in their zooming in and out. They also found that residents spent a larger share of their time at high magnification and needed more time for reaching their diagnoses than experts. Mello-Thoms et al. (2012) operationalised the efficiency of the diagnostic path in terms of stimulus coverage: the part of the image that was displayed per magnification range. They found that participants covered larger parts of the slide (at low, medium, and high magnification) when they, eventually, diagnosed the slide incorrectly. Incomprehension thus leads to a more intense search. Finally, Jaarsma et al. (2014) used static, two-dimensional images of tissue, allowing a fixed inspection time of 2 s. Eye movements in the first and second part of inspection time were compared. It was found that intermediates spent the second half of the inspection time in one area, while, conversely, the eye movements of experts were more dispersed in this second half. From these results it was concluded that intermediates, in the second half, tended to check the abnormalities they found in the first half to make sure they interpreted them correctly, while experts checked the rest of the image for anything else than they had already discovered.

Whereas characteristics of viewing behaviour are central to visual expertise research, cognitive expertise research focuses on the quantity, nature and structure of a diagnostician’s knowledge, as well as the reasoning strategies employed. The three-stage model of medical expertise development by Boshuizen and Schmidt is based on “the acquisition and development of knowledge structures upon which a student or a physician operates diagnosing a case” (Boshuizen and Schmidt 2008, p. 114). In the first stage, medical students construct knowledge networks of lower-order, biomedical concepts. Knowledge accretion and validation help them to create direct lines of reasoning between different concepts (ibid., pp. 114–115). When these lines of reasoning are activated repeatedly in the clinic, intermediate concepts are increasingly skipped to form higher-order concepts. This process of knowledge encapsulation allows the students to make direct links in their clinical reasoning, for example between a symptom and a diagnosis. In the last stage, so-called illness scripts take the place of the encapsulated knowledge network. Illness scripts are even more aggregated clusters of knowledge, including the enabling conditions for the pathophysiological fault, and the consequences of a specific disease.

Clinical reasoning is largely affected by the knowledge structure. In the first stage, the fine-grained conceptual network of a student requires long reasoning chains with small steps. Later on, knowledge encapsulations enable diagnosticians to directly link findings to hypotheses, cutting short of the long reasoning. The availability of illness scripts, in the last stage, allows expert diagnosticians to reason in the form of schemata, triggered by patient background or initial findings. As long as new findings correspond with this schema, no active reasoning is required (ibid., p. 115).

Apart from the knowledge structures, clinical reasoning is also studied from the perspective of the strategies applied by diagnosticians. Thirty years of research in this domain rendered the insight that experts apply multiple reasoning strategies, ranging from instant diagnosis based on pattern recognition, to the formulation and checking of hypotheses (i.e., the hypothetico-deductive model) (Schwartz and Elstein 2008). The application of the strategy depends on the familiarity with the case: familiar cases result in instant diagnoses, while unfamiliar ones demand the hypothetico-deductive approach. Intermediates and novices rely mainly on the latter approach. The quality of their hypotheses and their checking strategies are thereby lower than that of experts: they tend to gather more information to refute or confirm their hypotheses (Jensen et al. 2008).

From the above discussion of literature on visual and cognitive aspects of expertise of clinical pathologists, three constructs are derived: (1) encapsulations; (2) efficiency; and (3) hypothesis testing. Hypotheses are formed per construct. Both the constructs and hypotheses are described below (see also Table 1):

**Table 1:** Constructs, hypotheses, measures, and predictions per measure

Constructs	Hypotheses	Measures	Predictions
*Encapsulations* [Several small bits of information encapsulated in overarching visual constellations and cognitive concepts]	1. Diagnosticians with more expertise use more visual and cognitive encapsulations in their diagnoses than participants with less expertise	Average magnification	E < I < N
		Proportion of time per magnification range	Low: E = I = N
			Medium: E < I < N
			High: E < I < N
		Descriptives	E < I < N
		Specific pathologies	E < I > N
		Comparatives	E > I > N
*Efficiency* [The deliberate and goal oriented exploration of the slide]	2. The slide exploration of diagnosticians with more expertise is more goal-oriented than that of diagnosticians with less expertise	Panning movements	E < I < N
		Opposed zooming movements	E < I < N
		Stimulus coverage per magnification range	Low: E = I = N
			Medium: E < I < N
			High: E < I < N
		Time-to-first-hit of DRA	E < I < N
		Time on task	E < I < N
		Average fixation duration	E > I > N
		Average saccade length	E > I > N
		Reasoning terms	E < I < N
		Conclusives	E > I > N
*Hypothesis testing* [The collection of information for testing hypotheses]	3. Diagnosticians with high expertise use diagnostically relevant areas for confirmation, rather than for information as those with lower expertise do	Proportion of time in DRAs	E < I > N
		Number of DRAs visited	E < I > N
		Revisits to DRAs	E < I > N

*E* Experts, *I* Intermediates, *N* Novices

### **Hypothesis 1** (*Encapsulations*)

The concept of encapsulations applies to both cognitive and visual aspects of expertise. In a cognitive form, knowledge encapsulations will allow a diagnostician to verbalize findings in higher-order terms, as opposed to more detailed, low-level concepts. Visually, a certain feature viewed at low magnification transforms into a constellation of more detailed features at high magnification. The ability to interpret a visual encapsulation at low magnification is expected to depend on the level of expertise, just like knowledge encapsulations are not available yet to novices. Therefore, it is hypothesized that participants with higher expertise make more use of encapsulations (both visual and cognitive) than participants with lower expertise.

### **Hypothesis 2** (*Efficiency*)

Secondly, there is the efficiency of the diagnostic path. The previously discussed eye tracking studies revealed a more efficient scan path for experts, while the very few studies with microscope navigation data (Mello-Thoms et al. 2012; Treanor et al. 2009) showed a similar efficiency in this navigation. Also, knowledge structured in encapsulations and illness scripts will render more efficient reasoning: the reasoning chains of experienced diagnosticians will be shorter than those of less experienced task performers. In summary, it is hypothesized that diagnosticians with higher expertise will show more efficient diagnostic paths than those with lower expertise.

### **Hypothesis 3** (*Hypothesis testing*)

The third construct is hypothesis testing. The hypothetico-deductive model of clinical reasoning states that compared to experts intermediates and novices tend to gather more information to test their hypotheses. Also, in our previous study (Jaarsma et al. 2014), we have argued that intermediates tend to check their own findings by checking parts of the image they have already examined, whereas experts tend to check the remaining tissue on features that might change their tentative diagnosis. Therefore, it is hypothesized that compared to experts novices and intermediates collect more evidence to come to a diagnosis and tend to search for confirmation of their hypothesis rather than contrasting information.

Table 1 gives an overview of the constructs and the corresponding hypotheses and measures.

## Methods

### Participants and design

The participants in this study (*N* = 38, *M* = 35.39 years, *SD* = 14.67; 24 females) were recruited on a voluntary basis in two hospitals in the Netherlands. Based on their experience, they formed three expertise levels: Experts were 13 clinical pathologists (*M* = 51.77 years, *SD* = 10.05; 3 females) with an average experience of 21.38 years (*SD* = 10.03), including five years of training. Intermediates were 12 residents (*M* = 33.25 years, *SD* = 6.28; 8 females) with an average of three years of training (*SD* = 1.60). Novices were 13 s-year medical students who had completed two courses in the physiology and pathology of cells and tissue, including the gastrointestinal tract (*M* = 21.00 years, *SD* = 2.58, all females). All participants had good or corrected to good eyesight. They received a small gift for their participation (book voucher) after the experiment.

The experiment was set up as a between-subjects design, with expertise level as the independent variable. The sequence of the stimuli was based on a balanced Latin square.

The study was approved by the ethical committee of the Open University, who did not deem it to require any kind of formal ethical approval. All participants gave written informed consent.

### Materials and apparatus

#### Diagnostic task

The diagnostic tasks in the experiment all involved microscopic images of the colon, which were obtained from the Atrium Medical Centre in Heerlen, the Netherlands. A priori, these cases were diagnosed by four clinical pathologists and a consensus diagnosis was reached for all cases. The cases were divided over five diagnostic categories: adenocarcinoma (cancer, 2 cases), adenoma (pre-stage of cancer, 2 cases), inflammation (1 case), hyperplastic polyp (benign polyp, 1 case), and normal tissue (1 case).

To be able to record eye movements and the participants’ zooming and panning movements, the Aperio ImageScope digital microscope (version 11.2.780) was used, in combination with a 22-inch digital monitor (DELL P2210) with a resolution of 1,680 × 1,050 pixels. Using a digital microscope resembles navigating through a digital map: one can zoom in and zoom out, and pan horizontally. All these movements were thus recorded by the digital microscope for later analysis.

#### Recording of eye movements

Eye movements were recorded with the SMI RED eye tracker with a temporal resolution of 250 Hz and the SMI iViewX software (version 2.7.13). The presentation software of the same company was used to present patient background information and to collect the diagnoses (ExperimentCenter, version 3.2.11, www.smivision.com). In addition, the eye movement registration files were used to determine the time on task.

#### Participants’ background

A demographic questionnaire was used to collect background information on the participants, including sex, age, vision and experience with the colon and digital microscope. Additionally, the Miles’ test (Miles 1930) was used to determine the dominant eye, necessary for eye tracking data analysis.

### Procedure

The experiment was carried out in individual sessions of about thirty minutes. First, participants completed the demographic questionnaire and performed the Miles’ test for the dominant eye (Miles 1930). Then, they were introduced to the digital microscope and solved a pilot case to become acquainted with the microscope operation. The eye tracking system was calibrated for each participant and validated directly afterwards.

The setting simulated the normal working procedure. First, a written patient background was presented, including sex, age, kind of tissue, and comments or questions from the requesting physician. Then, participants entered the digital microscope and started the diagnostic process. As soon as they had come to a diagnosis, the participants closed the slide and pushed a button to proceed to select one out of five diagnostic categories: normal, adenoma, adenocarcinoma, inflammation, or hyperplastic polyp. Finally, the participants explained their choice verbally. This procedure was repeated for each case. The experiment ended with another validation of the eye tracker to control for offset.

### Data reduction and analysis

The experiment resulted in a total of 261 records of microscope navigation, 255 records of eye movements, and 215 verbal explanations. This section discusses the reduction and analysis of the data, and is therefore organised per data source. The measures derived from the data are discussed per construct.

#### Microscope navigation

The data recorded by the digital microscope consisted of basic information on the image parts that had been displayed, at which magnification and for how long. Accidental zooming actions (i.e., zooming actions followed within a second by a movement in the opposite direction) were deleted from the data. From these elementary data, several measures for the hypotheses were derived. We will discuss these per hypothesis.

##### Encapsulations

This construct deals with the extent to which diagnosticians used visual and cognitive encapsulations. Visual encapsulation was measured as the average magnification—a time-weighted average of all magnifications used by a participant in a single case—and the proportion of the time on task per magnification range. Three magnification ranges were identified as an independent variable: low (below 4×), medium (between 4× and 10×), and high (10× and beyond).^1^

##### Efficiency

Three measures were used to assess the efficiency of the diagnostic path. The number of panning movements gives the number of movements in the horizontal plane. The number of opposed zooming movements concerns the number of times the direction of magnification changes, e.g. from zooming out to zooming in. The stimulus coverage per magnification range (low, medium, and high) indicates how much of the actual image was viewed per magnification range. Finally, the time-to-first-hit in a diagnostically relevant area (relative to time on task) was used as an operationalization for how quick abnormalities were found. Diagnostically relevant areas (DRAs) were drawn on every slide by the third author and contained cues that were crucial for the correct diagnosis. Each case included several DRAs. It was considered a hit in one of these DRAs when the centre of the displayed part of the image fell within the DRA.

##### Hypothesis testing

This construct focuses completely on the examination of DRAs and was operationalised by the proportion of time on task spent in DRAs, number of DRAs visited (for each case), and revisits to DRAs.

#### Eye movements

The iViewX software recorded all eye movements, mouse clicks, keyboard strokes, and presentation times of stimuli. The time on task was determined by taking the difference in time between the keyboard stroke or mouse click opening the stimulus, and the one closing it. All eye movements in this period were considered as belonging to that specific case. Fixations were identified from these eye movements based on the velocity of eye shifts: periods when the velocity was less than 40 visual degrees per second were marked as fixations (with a minimum duration of 50 ms). Anything else was a saccade. Measures based on eye movements were formed only for the construct of efficiency. This efficiency was operationalised by time on task, average fixation duration (duration of the time an eye stays in one position), and average saccade length (distance between two fixations).

#### Clinical reasoning

The clinical reasoning was studied through a lexical analysis of the post hoc explanations of the diagnostic category that was chosen by the participants. The audio data were transcribed by a research assistant, and these transcripts were checked for correctness and completeness by the first author. The vast majority of these transcripts had a length between 10 and 100 words: a few were shorter or longer. The same coding categories were used as in Jaarsma et al. (2014), see Table 2. The categories that fitted one of the constructs will be discussed below.

**Table 2:** Categories of words used for the analysis of the verbal data, including a description and examples

Category	Description	Examples (words)
Reasoning	Words characteristic for a chain of reasoning	Because, as, so, but
Comparatives	Qualifications based on comparisons with mental models	Irregular, normal, increase
Conclusives	Words used to come to a conclusions	Diagnosis, characteristic (for)
Descriptives	Terms used to describe features in images in terms of colour and size	Purple, pink, round, flowers
Diagnosis	Words referring to a diagnosis	Adenoma, adenocarcinoma
Anatomy	Words referring to the anatomy of tissue and cells	Epithelium, nucleus, lamina propria
Pathologies	Specific terms referring to an anomaly in the tissue	Lymphocytes, invasion, infiltration
Overview	Words referring to overview of image and process	Architecture, coupe, patient information
Diagnostic specifications	Terms which specify a certain diagnosis	High-grade, infiltrating
Spatial orientation	Words referring to the spatial orientation of features in the image	Bottom, left, depth

##### Encapsulations

This construct was operationalised by the verbal categories of *descriptives*, *specific pathologies*, and *comparatives*. Descriptives represent the descriptions of features, such as colour, shape, or comparisons to everyday objects. These are lower-order concepts and associated with low expertise. Specific pathologies are biomedical terms for abnormalities, such as ‘lymphocytes’, ‘invasion’, and ‘infiltration’, which are higher-order concepts, or encapsulations, associated with higher expertise levels (intermediates, especially). Comparatives are direct links between findings and an indication of good/not good, such as (ab)normal and (a)typical. This last category, being very short linkages between findings and diagnoses (or diagnostic directions), is associated with high expertise.

##### Efficiency

It was argued that the characteristics of a knowledge structure influence clinical reasoning, especially in terms of efficiency. Therefore, we identified two verbal data categories indicative of the efficiency of clinical reasoning: *reasoning terms* and *conclusives*. Reasoning terms are all words used to stitch together lines of reasoning, such as ‘because’, ‘so’, and ‘but’. A high number of these words indicates long reasoning chains based on low-level concepts (e.g., findings). Conclusives, on the other hand, can be seen as short-cuts in reasoning. They include terms like ‘diagnosis:’ and ‘characteristic for:’.

## Results

All measures for the microscope navigation and eye movement data were analysed with multi-level analysis with expertise-level as a predictor, except for proportion of time per magnification range, stimulus coverage per magnification range, and revisits to DRAs, which were analysed with a Kruskal–Wallis test due to their non-normal distributions. The verbal data were analysed with a Chi square test, as the outcome and predictor were categorical (word category and expertise level, respectively). The given expected numbers of codes are based on the total number of codes per expertise level and per coding category.

### Encapsulations

Please see Table 3 for an overview of the outcomes of the variables related to the construct of encapsulations.

**Table 3:** Construct 1: encapsulations

	*p*	Novices			Intermediates			Experts			Pairwise	*p*
		Mean	SD	95 % CI	Mean	SD	95 % CI	Mean	SD	95 % CI		
*Microscope navigation*												
Average magnification	.07	5.86	.70	4.35–7.37	6.00	.71	4.48–7.52	4.80	.70	3.29–6.31	N–I	1.0
											N–E	.17
											I–E	.10
Proportion time at low magnification (%)	<.01	36.37	3.20	30.01–42.73	33.85	2.30	29.27–38.42	46.80	2.45	41.93–51.67	N–I	1.0
											N–E	<.01
											I–E	<.01
Proportion time at med. magnification (%)	<.01	42.45	2.80	36.88–48.01	33.26	2.02	29.24–37.28	25.38	2.26	20.87–29.86	N–I	.05
											N–E	<.01
											I–E	.05
Proportion time at high magnification (%)	.28	9.89	1.41	7.08–12.69	11.83	1.56	8.73–14.94	9.22	1.72	5.81–12.63	N–I	n.a.
											N–E	n.a.
											I–E	n.a.

	Novices			Intermediates			Experts		
	Count	Exp. Count	*p*	Count	Exp. Count	*p*	Count	Exp. Count	*p*
*Clinical reasoning*									
Descriptives	11	4	.001	0	4	.036	3	6	.271
Specific pathologies	35	66	<.001	96	70	.001	94	90	.689
Comparatives	33	48	.028	49	51	.764	84	66	.028

#### Microscope navigation

Expertise level was related to the proportion of time on task spent at low (*H*(2) = 16.19, *p* < .01) and medium (*H*(2) = 24.23, *p* < .01), but not at high magnification (*H*(2) = 2.57, *p* = .28). Pairwise comparisons (with Bonferroni correction) revealed that experts spent more time at low magnification than novices (*p* < .01, *r* = .27) and intermediates (*p* < .01, *r* = .25). At medium magnification, experts spent less time than intermediates (*p* = .05, *r* = .18) and novices (*p* < .01, *r* = .37). Intermediates spent less time in this range than novices (*p* = .05, *r* = .18).

There was a marginally significant effect of expertise level on the average magnification, *F*(2, 37.22) = 2.94, *p* = .07. Pairwise comparisons (with Bonferroni correction) showed there was a marginally significant difference between experts and intermediates (*b* = −1.20, *t*(37.37) = 2.19, *p* = .10), with a lower average magnification for experts than for intermediates.

#### Clinical reasoning

Expertise level was significantly associated with the frequencies of the kinds of terms used by our participants, χ^2^ (18, *N* = *1,478*) = 184, *p* < .01. To measure encapsulations, three categories were identified: comparative terms, descriptive terms, and specific pathologies. All three categories were typical for either one of the expertise levels: Experts used many comparative terms (*n* = 84, vs. 66 expected, *z* = 2.2, *p* = .03), intermediates used many specific pathologies (*n* = 96, vs. 70 expected, *z* = 3.2, *p* < .01), and novices used many descriptive terms(*n* = 11, vs. 4 expected, *z* = 3.4, *p* < .01).

### Efficiency

Please see Table 4 for an overview of the outcomes of the variables related to the construct of efficiency.

**Table 4:** Construct 2: efficiency

	*p*	Novices			Intermediates			Experts			Pairwise	*p*
		Mean	SD	95 % CI	Mean	SD	95 % CI	Mean	SD	95 % CI		
*Microscope navigation*												
Panning movements	.01	57.62	8.71	40.00–75.25	52.58	8.99	34.39–70.77	25.70	8.72	8.04–43.35	N–I	1.0
											N–E	.02
											I–E	.06
Opposed zooming movements	.01	6.77	1.02	4.70–8.84	8.40	1.06	6.25–10.56	3.98	1.03	1.90–6.06	N–I	.76
											N–E	.15
											I–E	.01
Stimulus coverage at low magnification (%)	.02	62.99	4.16	54.72–71.25	71.89	3.87	64.19–79.59	78.78	3.36	72.11–85.46	N–I	.14
											N–E	.02
											I–E	1.0
Stimulus coverage at med. magnification (%)	<.01	26.00	2.82	20.40–31.60	10.05	1.75	6.56–13.54	5.61	1.40	2.83–8.40	N–I	.04
											N–E	<.01
											I–E	.01
Stimulus coverage at high magnification (%)	.02	8.43	1.91	4.64–12.22	2.44	.74	.98–3.90	1.19	.52	.17–2.22	N–I	1.0
											N–E	.04
											I–E	.07
Time-to-first-hit in DRA (%)	.01	21.15	4.32	11.98–30.32	22.79	4.39	13.50–32.09	34.58	4.34	25.37–43.79	N–I	1.0
											N–E	.02
											I–E	.05
*Eye movements*												
Time on task (s)	.04	152	18.9	114–190	110	19.6	70–149	86	18.9	48–125	N–I	.31
											N–E	.04
											I–E	1.0
Average fixation duration (ms)	.06	284	12.11	259–309	245	10.91	223–267	257	11.18	235–280	N–I	.06
											N–E	.34
											I–E	1.0
Average saccade length (pixels)	.60	157	4.77	147–166	151	4.69	141–160	153	4.80	143–162	N–I	n.a.
											N–E	n.a.
											I–E	n.a.

	Novices			Intermediates			Experts		
	Count	Exp. Count	*p*	Count	Exp. Count	*p*	Count	Exp. Count	*p*
*Clinical reasoning*									
Reasoning terms	159	97	<.001	63	102	<.001	109	132	.046
Conclusives	3	22	<.001	39	24	.001	34	30	.484

#### Microscope navigation

Expertise level significantly predicted the participants’ time on task: *F*(2, 37.89) = 4.95, *p* = .01. Pairwise comparisons of expertise levels revealed a significantly shorter time on task for experts than for novices (*b* = −76.96, *t*(37.91) = −3.13, *p* = .01). No significant difference was found between experts and intermediates (*p* = .62), or between intermediates and novices (*p* = .25). Also, expertise level significantly predicted both the number of panning movements (F(2, 37.81) = 5.03, p = .01) and the number of opposed zooming movements (F(2, 38.16) = 5.03, p = .01). Pairwise comparisons showed that experts made significantly fewer panning movements than novices (*b* = −31.93, *t*(37.77) = −2.96, *p* = .02), marginally significantly fewer than intermediates (*b* = −26.88, *t*(37.92) = −2.44, *p* = .06), and also fewer opposed zooming movements than intermediates (*b* = −4.42, *t*(38.33) = −3.12, *p* = .01). The stimulus coverage per magnification range was affected by expertise level at low magnification (*H*(2) = 7.95, *p* = .02), medium magnification (*H*(2) = 30.04, *p* < .01), and high magnification (*H*(2) = 7.52, *p* = .02). Pairwise comparisons showed that experts covered a larger part of the tissue at low magnification than novices (*p* = .02, *r* = .20). At medium magnification, experts covered less of the image than intermediates (*p* = .01, *r* = .22) and novices (*p* < .01, *r* = .41). Intermediates covered less than novices (*p* = .04, *r* = .19). At high magnification, experts covered significantly less than novices (*p* = .04, *r* = .18), and marginally significantly less than intermediates (*p* = .07, *r* = .17).

As for the time-to-first-hit in a diagnostically relevant area (DRA), there was also a significant effect of expertise level, *F*(2, 36.39) = 5.10, *p* = .01. Pairwise comparisons revealed that experts entered a DRA later than both intermediates (*b* = 11.79, *t*(36.44) = 2.55, *p* = .05) and novices (*b* = 13.43, *t*(37.10) = 2.94, *p* = .02).

#### Eye movements

There was a marginally significant effect of expertise level on the average fixation duration, *F*(2, 61.71) = 2.91, *p* = .06. Pairwise comparisons exposed a marginally significant difference between intermediates and novices, with a shorter average fixation duration for intermediates (*b* = −39.32, *t*(41.43) = −2.41, *p* = .06).

There was no significant effect of expertise level on the average saccade length, *F*(2, 45.22) = .51, *p* = .60.

#### Clinical reasoning

Experts (*n* = 109, vs. 132 expected, *z* = −2, *p* = .05) and intermediates (*n* = 63, vs. 102 expected, *z* = −3.9, *p* < .01) used relatively few reasoning terms, whereas novices used relatively many of these terms (*n* = 159, vs. 97 expected, *z* = 6.4, *p* < .01). Conclusives were seldom used by novices (*n* = 3, vs. 22 expected, no *z*-value due to low score), while intermediates used them relatively often (*n* = 39, vs. 24 expected, *z* = 3.2, *p* < .01).

### Hypothesis testing

Please see Table 5 for an overview of the outcomes of the variables related to the construct of hypothesis testing.

**Table 5:** Construct 3: hypothesis testing

	*p*	Novices			Intermediates			Experts			Pairwise	*p*
		Mean	SD	95 % CI	Mean	SD	95 % CI	Mean	SD	95 % CI		
*Microscope navigation*												
Proportion of time spent in DRAs (%)	.20	41.29	7.78	21.73–60.84	44.52	7.81	24.97–64.08	39.52	7.79	19.96–59.07	N–I	n.a.
											N–E	n.a.
											I–E	n.a.
Number of DRAs visited	.03	4.05	.63	2.57–5.54	3.98	.64	2.50–5.47	3.05	.63	1.57–4.54	N–I	1.0
											N–E	.05
											I–E	.09
Revisits to DRAs	.09	2.90	.35	2.21–3.60	2.75	.34	2.07–3.43	2.08	.33	1.43–2.73	N–I	n.a.
											N–E	n.a.
											I–E	n.a.

#### Microscope navigation

Expertise level significantly affected the number of DRAs visited, *F*(2, 37.45) = 3.77, *p* = .03. Pairwise comparisons showed that experts visit marginally significantly fewer DRAs than both intermediates (*b* = −.93, *t*(37.90) = −2.25, *p* = .09) and novices (*b* = −1.00, *t*(37.35) = −2.48, *p* = .05). There was a marginally significant effect of expertise level on the number of revisits to DRAs (*H*(2) = 4.90, *p* = .09), but no significant effect of expertise level on the proportion of time on task spent in DRAs (*F*(2, 181.01) = 1.65, *p* = .20).

## Discussion

In the introduction to this article, three hypotheses were formed along three constructs derived from literature. Hypothesis 1 stated that diagnosticians with higher expertise use more visual and cognitive encapsulations in their diagnoses than diagnosticians with lower expertise. Expertise was predicted to correlate negatively with magnification. Besides, participants with higher expertise use more terms that combine findings with diagnoses or diagnostic directions, whereas participants with lower expertise would verbalize their thoughts using more detailed terms.

Most of these predictions were confirmed. Experts spent more of their time at low magnification than novices and intermediates, while they spent less time at medium magnification. Also, the average magnification of experts was marginally lower than that of intermediates. In their clinical reasoning, experts indeed used more comparative terms, with which they interpret findings in terms of normal/abnormal or typical/atypical. Meanwhile, intermediates expressed themselves more in terms of specific pathologies, showing a lower degree of using encapsulations. Novices, on the other end, used many descriptive terms, expressing themselves in colours and shapes.

Hypothesis 2 concerned the efficiency of the diagnostic path and stated that diagnosticians with higher expertise are more efficient than low expertise diagnosticians. More specifically, the predictions included shorter time on task, fewer horizontal and vertical movements, and smaller stimulus coverage for higher expertise participants. Eye movements would include longer fixations and longer distances between them. Finally, experts would have shorter lines of reasoning.

Many of these predictions were confirmed, indicating that experts were indeed more efficient in their diagnostic paths than intermediates and novices. Remarkable findings for this construct included the high number of opposed vertical movements of the intermediates. Apparently, they switched between zooming in and zooming out rather often. This could mean that many abnormalities were detected at low magnification, and many of them were checked at high magnification. The fact that intermediates were less efficient on this aspect of microscope navigation than both novices and experts, could hint at an intermediate effect (Schmidt and Boshuizen 1993). Also, the lack of significant results of eye tracking variables is noteworthy: there was no significant effect of expertise on average fixation duration and saccade length. This might be an effect of the nature of the stimuli used in this experiment, being zoomable (i.e., changeable, interactive) microscopic images. We will discuss the effect of stimuli in more detail later on in this discussion. Another interesting outcome was the experts’ relatively late visit to diagnostically relevant areas. This could be explained by their tendency to spend more time at low magnification at the start of the diagnostic process (e.g., to create an overview for the case at hand). An alternative explanation could be that there was a minimal magnification level for this variable, to exclude unintended fixations in DRAs at low magnifications. As experts used in general lower magnifications, this precaution may have caused a relatively late entry time into these areas.

Hypothesis 3 stated that low-expertise diagnosticians would collect more evidence for their diagnoses. This hypothesis was operationalised by identifying three measures on the behaviour regarding diagnostically relevant areas (DRAs). There were very few significant effects of level of expertise for this construct: Experts visited fewer DRAs than intermediates and novices, but all participants spent a similar proportion of their time in them and revisited them equally often. As only one prediction is confirmed by these results, this hypothesis cannot be confirmed. However the finding that experts visit fewer DRAs resonates with the finding of selective data collection by experts (e.g., Elstein, Shulman, and Sprafka (1979). It might indicate that experts need less further evidence for their diagnosis.

## Conclusions and implications

The first and foremost aim of this article was to study both visual and cognitive aspects of expertise in the diagnosis of medical images, and to find similarities between the two. A first observation is that the constructs of magnification and efficiency are applicable to both visual and cognitive expertise. A novice takes in detailed bits of information, such as colours and shapes, gathered through the examination of the image at high magnification. As a result, their microscopic scan paths are long and inefficient. This same inefficiency is apparent in cognitive phenomena: reasoning chains of novices are long, as their knowledge consists mainly of detailed concepts. Experts, then, rely on efficient methods for their diagnosis: high magnifications and few movements are used to quickly qualify tissue in terms of normal/abnormal. Put differently, the information gathered by the diagnosticians seems to be a reflection of their knowledge structures. The development of both aspects of expertise seems to be a process of ongoing integration. Before one correctly diagnoses images at low magnification, one must be sure what it includes at high magnification. This parallels with the theory on knowledge encapsulation by Boshuizen and Schmidt (2008), where the encapsulation (low magnification) follows the network of detailed concepts (high magnification).

Whereas previous studies on expertise of clinical pathologists restricted the complexity of the task (e.g., Krupinski et al. 2006; Tiersma et al. 2003), or of the data collected (e.g., Crowley et al. 2003; Mello-Thoms et al. 2012; Treanor et al. 2009), this study used an authentic task and collected data from three sources (eye movements, microscope navigation, and verbal protocols) to obtain a complete and extensive account of visual medical expertise. The combined registration of eye movements and microscope navigation had some implications for the data analysis. The interactive nature of the microscopic images cancelled the correspondence between a certain location on the screen and certain content of the image. Two subsequent fixations on the very centre of the screen could take up different bits of information in the case of an intermediate panning movement. This interplay between eye movements and navigation by hand could be the reason why results on eye movement measures from previous studies using static images were not replicated. As a result, the added value of eye movements in a study with interactive images could be debated: the data on the participants’ microscope navigation also gives a clear insight into what parts of the image are viewed. In this study, we therefore primarily used the navigation data to study visual expertise.

The combination of eye tracking and navigation data exposed another—not yet studied—aspect of expertise of clinical pathologists: eye-hand coordination. Being able to interact with the images, we assumed that the participants would move interesting areas to the centre of the computer screen to examine them (this was supported by observations during the experiment). Hence, we figured that participants might keep focused on the centre of the screen, and let the image pass this centre. So, boldly stated: we expected not the eye but the image to move. A crucial skill for this is eye-hand coordination, and it could well be argued that experts are better at this than non-experts as they have practiced this skill throughout their careers. However, preliminary analyses of the dispersion of experts’ eye movements did not render any significant result. This could be because the operation of the digital microscope used in this experiment differed from the light microscope our participants were acquainted with. These analyses were therefore not included in this study. However, the inclusion of eye-hand coordination as an aspect of clinical pathologist expertise is an interesting challenge for future research.

This study is the first one to include a quantitative analysis of microscope navigation data. This analysis adds new insights to the knowledge on clinical pathologists’ expertise. First of all, it shows different preferences for magnification level of diagnosticians with different levels of expertise. Secondly, the analysis of microscope navigation data reveals switches between several magnification levels, and therefore between modes of examination such as global versus focal viewing. This behaviour corresponds with the two-staged models described in literature (Kundel et al. 1978; Swensson 1980), be it that there is not necessarily one switch between the stages in the diagnostic process, but, especially for residents, several switches. On a practical level, this information is important because residents in clinical pathology (the intermediates in this study) receive daily in-house training from expert clinical pathologists. Insights in how residents and expert pathologist differ in their examination of images could help both parties to improve this training.

A couple of limitations of this study need mentioning. First of all, the majority of our participants was female, especially among the intermediates and novices. Although there is no evidence in literature to assume that female diagnosticians possess different visual medical expertise than male diagnosticians, a gender effect cannot be rejected by our results. Secondly, there was a selective loss of verbal explanations: although they were equally divided over the cases, fewer of these explanations were collected among intermediates and novices as compared to experts. Some participants kept forgetting to give these explanations and reminding them would hinder the collection of eye movement data (due to head movements). The fact that experts gave more explanations might be because the effort to do so was smaller for them but this would contradict other findings of expert explanations and think aloud protocols where it is commonly found that experts are less verbose. The statistical analysis used is rather robust and compensates for frequency differences by adapting the expected values. Yet, intermediates and novices may have selectively skipped the explanation of certain cases such as the more frustrating or boring ones. The analysis cannot detect such biases. A future study should be set up in such a way that protocols cannot be missed.

Concluding, this study provided several pieces of evidence to assume that vision and cognition are two components of the same construct: expertise. So far, most studies have focused on either one of the two kinds of expertise, despite the fact that both forms of expertise need to be intertwined to make a good diagnostician. This study is a first step towards such a holistic account of expertise in clinical pathology. With these and future insights, an overarching theory on the expertise of clinical pathologists can be developed, transcending the boundaries between visual and cognitive expertise.

## Footnotes

1 These ranges are based on (and similar to) those used by Mello-Thoms et al. (2012).

## References

[CR1] Boshuizen HPA, Schmidt HG (1992). On the role of biomedical knowledge in clinical reasoning by experts, intermediates and novices. Cognitive Science.

[CR2] Boshuizen HPA, Schmidt HG, Higgs J, Jones MA, Loftus S, Christensen N (2008). The development of clinical reasoning expertise. Clinical reasoning in the health professions.

[CR3] Crowley RS, Naus GJ, Stewart J, Friedman CP (2003). Development of visual diagnostic expertise in pathology—An information-processing study. Journal of the American Medical Informatics Association.

[CR4] Elstein AS, Shulman LS, Sprafka SA (1979). Medical problem solving: An analysis of clinical reasoning.

[CR5] Jaarsma, T., Jarodzka, H., Nap, M., Van Merriënboer, J. J. G., & Boshuizen, H. P. A. (2014). Expertise under the microscope: Processing histopathological slides. *Medical Education, 48*(3). 10.1111/medu.12385 24528464

[CR6] Jensen G, Resnik L, Haddad A, Higgs J, Jones MA, Loftus S, Christensen N (2008). Expertise and clinical reasoning. Clinical reasoning in the health professions.

[CR7] Krupinski EA, Graham AR, Weinstein RS (2013). Characterizing the development of visual search expertise in pathology residents viewing whole slide images. Human Pathology.

[CR8] Krupinski EA, Tillack AA, Richter L, Henderson JT, Bhattacharyya AK, Scott KM (2006). Eye-movement study and human performance using telepathology virtual slides: Implications for medical education and differences with experience. Human Pathology.

[CR9] Kundel HL, Nodine CF, Carmody D (1978). Visual scanning, pattern recognition and decision-making in pulmonary nodule detection. Investigative Radiology.

[CR10] Mello-Thoms C, Mello CAB, Medvedeva O, Castine M, Legowski E, Gardner G (2012). Perceptual analysis of the reading of dermatopathology virtual slides by pathology residents. Archives of Pathology and Laboratory Medicine.

[CR11] Miles WR (1930). Ocular dominance in human adults. The Journal of General Psychology.

[CR12] Reingold EM, Sheridan H, Liversedge S, Gilchrist I, Everling S (2011). Eye movements and visual expertise in chess and medicine. The Oxford handbook of eye movements.

[CR13] Schmidt HG, Boshuizen HPA (1993). On the origin of intermediate effects in clinical case recall. Memory and Cognition.

[CR14] Schwartz A, Elstein AS, Higgs J, Jones M, Loftus S, Christensen N (2008). Clinical reasoning in medicine. Clinical reasoning in the health professions.

[CR15] Swensson R (1980). A two-stage detection model applied to skilled visual search by radiologists. Perception and Psychophysics.

[CR16] Tiersma ESM, Peters AAW, Mooij HA, Fleuren GJ (2003). Visualising scanning patterns of pathologists in the grading of cervical intraepithelial neoplasia. Journal of Clinical Pathology.

[CR17] Treanor D, Lim CH, Magee D, Bulpitt A, Quirke P (2009). Tracking with virtual slides: a tool to study diagnostic error in histopathology. Histopathology.

